# Hijacking PrP^c^-dependent signal transduction: when prions impair Aβ clearance

**DOI:** 10.3389/fnagi.2014.00025

**Published:** 2014-02-28

**Authors:** Julia Hernandez-Rapp, Séverine Martin-Lannerée, Théo Z. Hirsch, Jean-Marie Launay, Sophie Mouillet-Richard

**Affiliations:** ^1^INSERM UMR-S1124Paris, France; ^2^Sorbonne Paris Cité, UMR-S1124, Université Paris DescartesParis, France; ^3^Université Paris Sud 11, ED419 BiosigneOrsay, France; ^4^AP-HP Service de Biochimie, Fondation FondaMental, INSERM U942 H ôpital LariboisièreParis, France; ^5^Pharma Research Department, F. Hoffmann-La-Roche Ltd.Basel, Switzerland

**Keywords:** cellular prion protein, prion infection, Aβ clearance, signal transduction, Alzheimer’s disease

## Abstract

The cellular prion protein PrP^c^ is the normal counterpart of the scrapie prion protein PrP^ Sc^, the main component of the infectious agent of transmissible spongiform encephalopathies. The recent discovery that PrP^ c^ can serve as a receptor for the amyloid beta (Aβ) peptide and relay its neurotoxicity is sparking renewed interest on this protein and its involvement in signal transduction processes. Disease-associated PrP^ Sc^ shares with Aβ the ability to hijack PrP^ c^-dependent signaling cascades, and thereby instigate pathogenic events. Among these is an impairment of Aβ clearance, uncovered in prion-infected neuronal cells. These findings add another facet to the intricate interplay between PrP^ c^ and Aβ. Here, we summarize the connection between PrP-mediated signaling and Aβ clearance and discuss its pathological implications.

## INTRODUCTION

Up to recent years, commonalities between the amyloid precursor protein (APP) and the cellular prion protein PrP^c^ were considered to mainly reside in their capacity to give rise to aggregation-prone proteins, amyloid beta (Aβ) and PrP^Sc^ (standing for scrapie isoform of the prion protein), both involved in neurodegenerative disorders, Alzheimer’s disease (AD) and transmissible spongiform encephalopathies (TSEs), respectively (Haass and Selkoe, 2007; Aguzzi and Calella, 2009). These two diseases share neuropathological features, including synaptic damage, neuronal loss and astrogliosis (Reiniger et al., 2011). As for AD, human prion diseases may have a genetic origin, while most cases are sporadic (Aguzzi and Calella, 2009). Sporadic human prion diseases are thought to arise from the spontaneous conformational conversion of PrP^c^ into its pathogenic PrP^Sc^ counterpart (Aguzzi and Calella, 2009). PrP^Sc^ has a propensity to aggregate, form amyloid-like structures and can act as a seed to transmit its aberrant conformation to native PrP^c^ molecules (Aguzzi and Calella, 2009). Such template-directed misfolding is also now established in the case of Aβ species [(Jucker and Walker, 2013) for review]. Like Aβ (Langer et al., 2011), much evidence suggests that PrP^Sc^-associated toxicity is imparted by small oligomers (Silveira et al., 2005). This toxicity is assumed to be driven by the subversion of the normal function of PrP^c^ (Harris and True, 2006), which now also emerges as a key event in Aβ-induced neuronal damage (Lauren et al., 2009). The discovery that PrP^c^ may serve as a receptor for Aβ is calling for a better understanding of the role played by PrP^c^ in neurons. The relationship between Aβ and PrP^c^ actually extends beyond that of a ligand-receptor connection, since the prion-induced subversion of PrP^c^-dependent signaling causes impaired Aβ clearance (Pradines et al., 2013). In this review, we summarize the recent advances focusing on the Aβ-PrP duo and discuss the ensuing challenges.

## THE BASICS OF PrP^c^

**FIGURE 1:**
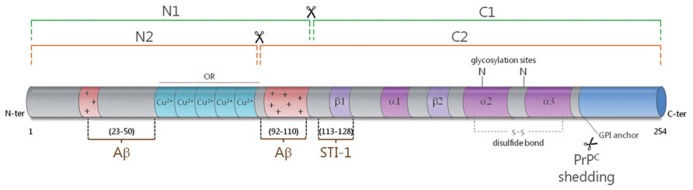
**Schematic representation of the secondary structure of PrP^c^.** The alpha-cleavage (green) occurs at position 111/112 and generates the N1 and C1 fragments. The beta-cleavage (orange) occurs in the vicinity of octapeptide repeats (ORs), which bind copper ions, and generates the N2 and C2 fragments. PrP^c^ can also be shed from the plasma membrane through the action of ADAM10. The protective factor STI-1 binds to PrP^c^ at amino acids 113–128. Two main binding sites have been mapped for Aβ to the very N-terminus (AA 23–50) and near the alpha-cleavage site at amino acids 92–110. The alpha helices (α) and beta sheets (β) are shown in purple and the polybasic regions (+) are shown in red.

Understanding the interplay between Aβ and PrP^c^ necessitates some focus on the latter protein. PrP^c^ is encoded by a unique gene, *Prnp*, whose open reading frame is contained within a single exon (Aguzzi and Calella, 2009). While ubiquitous, this protein is most abundantly expressed in neurons (Linden et al., 2008). It is located at the outer leaflet of the plasma membrane, to which it is attached through a glycosyl-phosphatidylinositol (GPI) moiety. PrP^c^ contains two potentially glycosylated aspargine residues, at the origin of a great diversity of isoforms (Ermonval et al., 2003). It can also undergo three types of proteolytical cleavages. The best studied are a so-called alpha-cleavage at position 111/112, yielding a N-terminal N1 fragment and a C-terminal C1 fragment, and a beta-cleavage in its N-terminal region giving rise to N2 and C2 fragments [reviewed in (Checler and Vincent, 2002), **Figure 1**]. The third cleavage leads to the release of a full-length anchorless isoform of PrP^c^ through the action of the metalloprotease ADAM10 (Altmeppen et al., 2011; **Figure 1**). Of note, similarly to APP processing, the alpha-cleavage of PrP^c^ occurs within a region bearing toxicity (residues 106 to 126), and may involve the TNF-alpha converting enzyme (TACE) metalloprotease (Checler and Vincent, 2002), although the nature of the proteases driving this alpha-cleavage is subject to controversy (Altmeppen et al., 2011; Beland et al., 2012). PrP^c^ thus exists under a plethora of isoforms, whose distribution may vary in distinct brain regions, cell types or even subcellular compartments and whose respective functions remain to be thoroughly resolved (Linden et al., 2008). Clues as to PrP^c^ normal function were greatly anticipated from the development of PrP-null mice. However, these mice are viable, develop normally and do not suffer from major abnormalities (Steele et al., 2007). Subtle behavioral and cognitive deficits were subsequently reported in PrP-deficient mice (Linden et al., 2008), in addition to an increased vulnerability to various types of stresses (Resenberger et al., 2011b). Noteworthy, these animals have been instrumental in substantiating the central role exerted by PrP^c^ in the development of prion diseases, since the knockout of the *Prnp* gene confers resistance to prion infection (Bueler et al., 1993). Further, post-infection knockdown of PrP^c^ in neurons is sufficient to counteract the progression of prion neuropathogenesis (Mallucci et al., 2003). Finally, transgenic mice expressing an anchorless variant of PrP^c^ (ΔGPI-PrP) were found to efficiently replicate prions upon infection, while not showing any sign of neurodegeneration (Chesebro et al., 2005). Overall, it is now quite well established that neuronal, GPI-anchored PrP^c^ serves as a relay of PrP^Sc^-induced neuropathogenesis. On this basis, understanding the normal function held by PrP^c^ in neurons appears as a necessary step to grasp how it is corrupted by its pathogenic counterpart, PrP^Sc^. Actually, this notion now also applies to Aβ-related pathogenesis, as will be emphasized below.

## PrP^c^-DEPENDENT SIGNALING IN NEURONS: FINDINGS AND IMPLICATIONS

The involvement of PrP^c^ in signal transduction was initially suspected from its cell surface location, as well as its enrichment in lipid rafts (Lewis and Hooper, 2011), which allow the spatial segregation of cell signaling components (Parton and del Pozo, 2013). Using a neuronal cell line (1C11) endowed with the capacity to differentiate into either serotonergic (1C11^5-HT^) or noradrenergic (1C11^NE^) neurons, we demonstrated that PrP^c^ has the capacity to trigger the activation of the Fyn kinase in neuronal cells (Mouillet-Richard et al., 2000). This signaling cascade is exclusively imparted by PrP^c^ molecules located on the neurites of differentiated cells and is relayed by the scaffold protein caveolin-1 (Mouillet-Richard et al., 2000). Downstream from Fyn, PrP^c^ can mobilize the reactive oxygen species (ROS) generating enzyme NADPH oxidase, and the transcription factors ERK1/2, cAMP response element-binding protein (CREB), Egr-1 and c-Fos (Schneider et al., 2003; Pradines et al., 2008). From a functional point of view, the PrP^c^-Fyn coupling was shown to mediate neural cell adhesion molecule (NCAM)-dependent neurite outgrowth (Santuccione et al., 2005) and to control calcium influxes in hippocampal neurons (Krebs et al., 2007). As for NADPH oxidase-derived ROS, they can promote the catalytic activation of TACE, subsequent TNFα shedding and bioamine catabolism, thus exerting a neuromodulatory function (Pradines et al., 2009). The demonstration that PrP^c^ could serve as a receptor for the chaperone protein STI-1 (Zanata et al., 2002) further set the stage for the identification of various cell signaling pathways involved in neuroprotection (Chiarini et al., 2002; Lopes et al., 2005), neurite outgrowth (Lopes et al., 2005), and/or memory consolidation (Coitinho et al., 2007). Interestingly, the contribution of PrP^c^ to some of these processes may involve additional partners, such as laminin (Coitinho et al., 2006; Beraldo et al., 2011; Santos et al., 2013). The PrP^c^-STI-1 interaction was also reported to sustain neuronal protein synthesis via the mTOR pathway (Roffe et al., 2010). Finally, multiple connections have been depicted between PrP^c^ and neurotransmitter signaling including crosstalk with serotonergic (Mouillet-Richard et al., 2005), nicotinic (Beraldo et al., 2010) and mostly glutamatergic receptors, whether metabotropic (Beraldo et al., 2011) or ionotropic (Stys et al., 2012). Collectively, there is now compelling evidence for the involvement of PrP^c^ in multiple signal transduction cascades, whose deviation may drastically impact on neuronal function and activity.

## PATHOGENIC PRIONS DEVIATE PrP^c^SIGNALING

**FIGURE 2:**
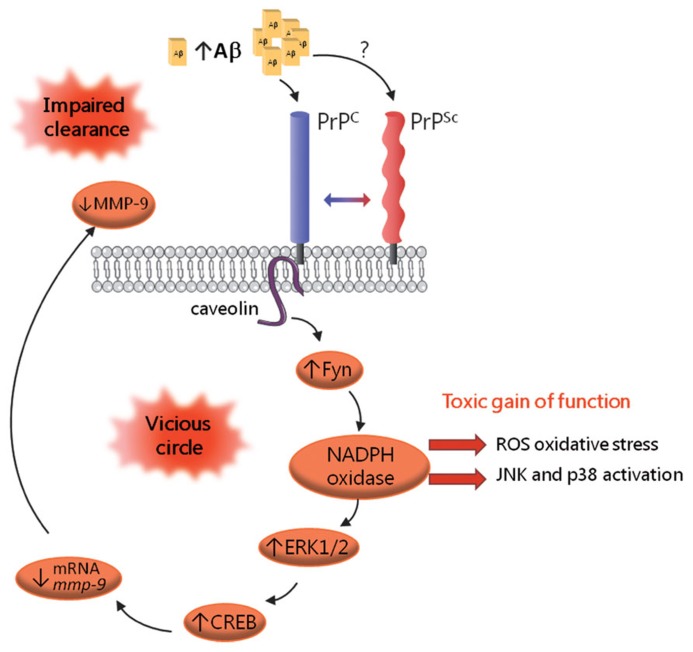
**Schematic representation of the corruption of PrP^c^-mediated signaling in prion-infected cells.** PrP^Sc^ accumulation is associated with the constitutive recruitment of various effectors of PrP^c^, including the Fyn kinase, the MAP kinases ERK1/2 and the CREB transcription factor. Prion-infected cells also exhibit recruitment of the stress-associated kinases p38 and JNK, which mirrors oxidative stress conditions. A downstream event in this cascade is the drastic reduction in MMP-9 mRNAs and activity, which, in turn, causes an impairment in Aβ clearance, leading to Aβ accumulation. Aβ oligomers would bind PrP^c^ and possibly PrP^Sc^ and further fuel the activation of this signaling cascade, thereby sustaining a vicious circle.

While it is now acknowledged that PrP^c^ is mandatory for the neurotoxic action of PrP^Sc^, our current understanding of how prions corrupt the physiological function of PrP^c^ is still far from complete (Harris and True, 2006). Achieving this goal is all the more challenging since PrP^c^ encompasses a great variety of isoforms, which may be associated with distinct partners and sustain promiscuous functions. In addition, the repertoire of PrP^c^ species eligible for *de novo* conversion into PrP^Sc^ may vary according to the prion strain and the cell type considered (Ermonval et al., 2003). Despite these hurdles, the development of *in vitro* modelsof prion infection has allowed to shed some light on this issue. Taking advantage of thecapacity of the 1C11 cell line to replicate various prion strains (Mouillet-Richard et al., 2008), we recently reported on the constitutive activation of signaling targets normally coupled with PrP^c^ in chronically infected cells (Pradines et al., 2013). In these cells as well as in prion-infected neurospheres, prion accumulation is associated with increased activities of Fyn, ERK1/2 and CREB (**Figure 2**). Another consequence of prion infection is the recruitment of the stress-associated kinases p38 and JNK, as a result of unbalanced ROS production. Downstream from CREB, we documented that prion-infected cells exhibit a reduced activity of the matrix metalloprotease MMP-9. This observation led us to delineate an impact on the clearance of the Aβ peptide, as will be discussed below. Collectively, these findings support the view that PrP^Sc^ exerts a toxic gain of PrP^c^ function. In line with this “gain of function” scheme, a central role of the NADPH oxidase NOX2 subunit in prion-induced neuronal damage was established using cerebellar organotypic culture slices (Falsig et al., 2012). Notwithstanding, some PrP^c^ functions may conversely be disrupted within an infected context. One such example is the loss of TACE activity, which renders cells highly sensitive to TNFα-induced cell death (Pietri et al., 2013). The “loss of function” hypothesis accommodates well with the neuroprotective activity ascribed to PrP^c^ (Resenberger et al., 2011b). Notably, the loss of PrP^c^ protective function within an infected context may relate to the inability of PrP^Sc^ to undergo proteolytic processing at position 111/112 (McMahon, 2012), and thereby generate the N1 fragment, endowed with neuroprotective activity (Guillot-Sestier and Checler, 2012). Most likely, prion-associated neuropathogenesis involves both gain and loss of function events, that may altogether promote synaptic dysfunction, oxidative stress, loss of neuronal homeostasis and ultimately neurodegeneration (Kovacs and Budka, 2008).

## PrP^c^TRANSDUCES Aβ TOXICITY

The first demonstration that PrP^c^ can bind Aβ came from the Strittmatter group in 2009, via an unbiased screening for receptors that could bind Aβ42 oligomers in the form of Aβ-derived diffusible ligands (ADDLs; Lauren et al., 2009). Lauren et al. (2009) mapped the interaction site to residues 92-110 in PrP^c^, i.e., the extremity of the N1 fragment (**Figure 1**), and showed that PrP^c^ supports about 50% of Aβ binding, in line with the multiplicity of Aβ receptors (Benilova and De Strooper, 2013). In that study, the impairment of synaptic plasticity fostered by ADDL was shown to be PrP^c^-dependent. While the involvement of PrP^c^ in Aβ-induced neurotoxicity was initially subject to controversy (Balducci et al., 2010; Calella et al., 2010; Kessels et al., 2010; Cisse et al., 2011), possibly as a result of distinct experimental paradigms, notably in the preparation of Aβ mixtures (Nicoll et al., 2013), all groups agreed that PrP^c^ has very good affinity for Aβ (within the nanomolar range). Over the past three years, the contribution of PrP^c^ as a relay of Aβ-dependent pathogenic effects has been corroborated and refined by several studies. For instance, targeting PrP^c^ using neutralizing antibodies was shown to counteract the Aβ-mediated impairment in synaptic plasticity (Barry et al., 2011; Freir et al., 2011; Kudo et al., 2012). Molecular analyses have further revealed that Aβ oligomers crosslink PrP^c^ to induce synaptic damage (Bate and Williams, 2011). As for PrP^Sc^, Aβ-induced toxic signaling via PrP^c^ requires its GPI-anchor, as well as lipid raft integrity (Resenberger et al., 2011a; Um et al., 2012; Rushworth et al., 2013). Besides, Aβ was shown to enhance the clustering of PrP^c^ at the cell surface of neuronal cells (Caetano et al., 2011). Taken together, these studies argue that Aβ oligomers amplify the duration or strength of PrP^c^ signaling. This scenario is also supported by several reports that have emphasized the corruption of the PrP^c^-Fyn signaling cascade by Aβ (Larson et al., 2012; Um et al., 2012, 2013). Um et al. (2013) were able to relate the Aβ-dependent subversion of the PrP^c^ coupling to Fyn to overactivation of the mGluR5 or NMDA receptor (Um et al., 2012), the latter observation being recapitulated in various other studies (Resenberger et al., 2011a; You et al., 2012). Taking a step further, Larson et al. (2012) demonstrated that the PrP^c^-Fyn complex promotes tau hyperphosphorylation in response to oligomeric Aβ, thus providing some molecular explanation for the well-established role of Fyn as a mediator of Aβ/tau-associated toxicity (Ittner and Gotz, 2011; Roberson et al., 2011). Whether the recruitment of the PrP^c^-Fyn complex by Aβ impacts on additional signaling effectors such as NADPH oxidase, similarly to PrP^Sc^ (Pradines et al., 2013), deserves further investigation. Finally, the recent demonstration that the binding of Aβ to PrP^c^ and the ensuing toxic events can be efficiently inhibited by the PrP^c^ alternate ligand STI-1 is providing a tangible avenue to disrupt the deleterious Aβ-PrP^c^ interaction (Ostapchenko et al., 2013).

## MULTIFACETED CONTROL OF PrP^c^ ON Aβ PRODUCTION AND AVAILABILITY

Aβ is generated from APP through the amyloidogenic processing pathway, which involves the beta-secretase BACE1 and occurs in a mutually exclusive fashion with the non-amyloidogenic pathway largely controlled by the alpha-secretase TACE (Checler and Vincent, 2002). In 2007, the Hooper group reported that PrP^c^ decreases the amyloidogenic processing of APP, thereby decreasing Aβ levels, a property that is lost in the context of prion infection (Parkin et al., 2007). Griffiths et al. (2011) went on to show that PrP^c^ interacts with BACE1 and controls its subcellular compartmentation. Of note, while this spatial control is protective toward the cleavage of wild-type APP, it does not prevent BACE1 from processing the Swedish mutant form of APP (Griffiths et al., 2011). Adding yet another layer of complexity to the picture, PrP^c^ also regulates the activity of TACE, a function that is deviated in an Alzheimer’s context and causes an imbalance of APP cleavage toward the amyloidogenic pathway (Pietri et al., 2013). PrP^c^ also regulates the availability of Aβ at several levels. For instance, PrP^c^ could favor the accumulation of Aβ in the brain by contributing to its transcytosis across the blood brain barrier (Pflanzner et al., 2012). Finally, in accordance with the binding of Aβ to various sites located within the N-terminal region of PrP^c^ (Chen et al., 2010; see **Figure 1**) and with the physiological processing of PrP^c^ at position 111/112, the resulting PrP^c^-derived N1 fragment was reported to trap Aβ and thereby exert a protective action against Aβ-induced cell death (Beland et al., 2012; Guillot-Sestier et al., 2012).

## HIJACKING PrP^c^ SIGNALING BY PATHOGENIC PRIONS CAUSES IMPAIRED Aβ CLEARANCE

As mentioned above, we documented that the subversion of PrP^c^ signaling by PrP^Sc^ in prion-infected cells leads to decreased activity of the MMP-9 metalloprotease (Pradines et al., 2013; **Figure 2**). MMP-9 exhibits alpha-secretase activity (Fragkouli et al., 2012) and also features among the Aβ degrading enzymes [(De Strooper, 2010) for review]. On this basis, we examined the potential outcome of the prion-dependent reduction in MMP-9 activity on Aβ metabolism. By assessing the production and elimination rates of Aβ peptides in prion-infected 1C11 cells, we substantiated an imbalance in the clearance of Aβ under prion infection, which was canceled after knockdown of the Fyn kinase (Pradines et al., 2013; **Figure 2**). We further observed a similarly impaired Aβ clearance in uninfected cells upon inhibition of MMP-9, highlighting the involvement of MMP-9 in this process. In agreement, we found increased levels of Aβ in the supernatants of prion-infected 1C11 cells or MMP-9 inhibitor-treated non-infected cells. Besides, we monitored an increase in the cerebrospinal fluid (CSF) levels of Aβ in mice treated with a MMP-9 inhibitor as well as in mice inoculated with prion-infected 1C11 cells. Altogether, these results delineate a reciprocal connection between PrP and Aβ that may sustain a pathogenic loop. Indeed, by promoting an accumulation of Aβ in the extracellular space, prion infection would fuel the recruitment of Fyn and its downstream targets through the interaction of Aβ with PrP, whether in its cellular or aggregated form (Chen et al., 2010; Zou et al., 2011). Whether the subversion of PrP^c^ dependent signal transduction by Aβ in Alzheimer’s disease also negatively impacts on MMP-9 activity and thereby impinges on Aβ clearance remains to be investigated. Should it be confirmed, disrupting this vicious circle would appear as a promising avenue to combat both prion and Alzheimer’s neuropathogenesis.

## CONCLUDING REMARKS

As outlined above, tremendous progress has been achieved in our understanding of the Aβ-PrP^c^ interplay in very recent years. The bidirectional relationship between these two proteins in signal transduction cascades may account for some -once puzzling- observations that deposits of Aβ and PrP may co-occur in patients with mixed AD and CJD clinical manifestation (Muramoto et al., 1992; Hainfellner et al., 1998; Debatin et al., 2008). Beyond the various PrP^c^-Aβ connections summarized above, the possibility that the interaction between the two proteins may favor their aggregation in a “cross-seeding” fashion is beginning to be explored (Morales et al., 2010). Overall, the more we learn in the field, the more we appraise the complexity of the PrP^c^-Aβ relationship. The latest advance has further extended the points of convergence in the molecular pathogenic pathways at play in the two diseases, including loss of TACE activity (Pietri et al., 2013) and impairment of the unfolded protein response (Moreno et al., 2012; Ma et al., 2013). Striking differences however remain between the two disorders, most notably the infectious properties specific to prions (Jucker and Walker, 2013). The only partial overlap between the two pathologies may originate from the extremely diverse array of PrP^c^ and Aβ species as well as their promiscuous partners (Linden et al., 2008; Benilova et al., 2012). Notwithstanding, the central role ascribed to PrP^c^ in defined Aβ-related toxic pathways opens new avenues for therapeutic intervention in AD.

## Conflict of Interest Statement

The authors declare that the research was conducted in the absence of any commercial or financial relationships that could be construed as a potential conflict of interest.
